# Recruitment process that attracts underrepresented students in graduate science and medical education programs

**DOI:** 10.1080/10872981.2021.1959283

**Published:** 2021-08-05

**Authors:** Titus A. Reaves, Meghan Anderson-Thomas

**Affiliations:** Department of Regenerative Medicine and Cell Biology, Medical University of South Carolina, Charleston, SC, USA

**Keywords:** Biomedical workforce, medical and science education, underrepresented groups, college, k12

## Abstract

While the population of minorities is increasing in the USA, the numbers obtaining advanced degrees in science/engineering and medicine are minimal. Underrepresented groups make up 19% of the USA labor pool, but less than 6% of science (engineering and medicine) Ph.D.’s. Diversifying the universities and health-care institutions is important to improve the academic experience of faculty, staff, students and everyone regardless of race. To prepare for the approaching diverse environments, educational institutions must create programs that allow underrepresented groups thrive in higher education; and logistically to be sustaining, the recruitment programs must begin at the student level. One approach is the integration of the history (the linking of past-heroes with present-heroes) of science; which is an interesting and important paradigm that can be implemented. As such, a day-long symposium highlighting the life and accomplishments of an African American Scientist; Dr. Ernest Everett Just, Ph.D., is used as a working model to inspire, educate on admission requirements, and to recruit into graduate science and medical programs.

## Introduction

Necessity for a Diverse Biomedical Workforce

It has been revealed from several reports that the population of minorities is increasing in the USA, but the numbers obtaining advanced degrees in science/engineering and medicine are minimal [1,2]. The importance of a diverse health care and biomedical workforce cannot be understated [3]. Evidence indicates that multiculturalism in universities and health-care organizations is not only associated with improved access to health care for racial and ethnic patients, but there is greater patient satisfaction and better educational experiences for all health professionals and patients regardless of race or ethnicity [4,5]. When information and health care are received from a health-care official who is racially similar, the comfort level of patients is increased and access to health care is improved [6, 7, Earl, 2013 #177; 5]. When that same information and health care is received from non-underrepresented minority official, it is viewed as educational [5]. This is not to suggest that only racially similar health-care personnel can treat racially similar patients. However, not seeing any racially similar health-care officials (from nursing technician to physician) may affect overall health of the patient.

The rapidly approaching multiculturalism in the population of the USA necessitates that medical education examines the effects of diversity on biomedical research and health care. [8, 9,#366, #196; 3, 5, 10–14]. Projections based on the U.S. census data indicate that for the period 2020–2060, the US population is expected to grow by nearly 79 million with minority (Hispanic, African American and mixed races populations) being the fastest [15]. This situation strongly reveals that we need Black/African Americans and Hispanic/Latino with education and training in science and medicine [2,11]. To prepare, institutions are taking aggressive steps to maximize the diversity of academic health professionals, administrators, and health-care providers who participate in quality health-care initiatives and programs [16, 17]. At the University of the District of Columbia, they offer a new Bachelor of Science in Biomedical Engineering. The educational goal of this program is to enhance the multiculturalism of the biomedical workforce via student recruitment and professional development [16]. Enrolling students in science as undergraduates may lead to an increase in graduate education matriculation [16]. The Medical School at UT Health in Houston, Texas uses a holistic approach to recruitment of residents. For example, while there must be a baseline, selection criteria goes beyond academic metrics. They identify student stakeholders, core faculty, and residents to act as representatives on the program evaluation committee, all of which is guided by the medical school’s mission to train diverse physicians, care for the underserved, and to reduce\eliminate health-care disparities [18].

The first step is to recognize that role model underrepresented scientists and faculty members in medical schools are not proportional to the changing population demographics and warrants the recruitment at the student level to generate a long-term diverse biomedical workforce. Recruiting this diverse workforce will require that university enrollment strongly resemble demographics of the state and the country. At the Medical University of South Carolina (MUSC), we have developed the Ernest Everett Just symposium as an innovative method for the recruitment and education of underrepresented students in the sciences and medicine. In addition, we also provide outcomes from 2013 to 2019 in terms of attendance, interest in science and medicine as a career, and recruitment into the graduate and science programs. The purpose of this manuscript is to describe and address the need for innovative programs that deal with educational disparities, provide an overview of the life of Dr. Just, and outline the design of this program, which could serve as an innovative model that can be duplicated at other institutions.

### Inspiring/educating/recruiting: the Ernest Everett Just symposium as a model that recruits a diverse biomedical workforce

Reasons for the disparity in the biomedical workforce in relation to the general population are multifactorial. First, compared to previous generations and regardless of race, the US does not produce a high proportion of engineers, scientists or mathematicians [2,19,20]. Thus, fewer are becoming committed to STEM careers [2]. Second, increases in income of other professions that only require a 4-year undergraduate degree makes graduate/professional school less attractive [2]. Third, from our experience many underrepresented students lack information on how to pursue careers that require a graduate degree [21,22]. This lack of information includes funding mechanisms, course prerequisites, letters of recommendations, and how to perform hypothesis-driven bench science [4, 21, 22,#419]. Fourth, students need better mentors that will direct them to be high achievers and to seek careers that require graduate science and medical degrees [23–25]. It is clear that there are not enough underrepresented faculty to mentor underrepresented students. However, faculty do not have to look like the student to mentor – they just need to take interest. However, they do need to motivate and inspire the student [26].

Lastly, while family members have tremendous pride in the acceptance letters of their children, the absence of the experience in the rigors of graduate science and medicine curricula makes their prospective contribution less influential (i.e., they do not know how to help) [21, 22,#419]. Parents are encouraged to expose their children to after-school programs in science, visit research laboratories at local universities to learn how research is generated so that they may receive instruction on how to organize research results in a manuscript that can be published [21,22]. It would also be beneficial if science and medical program directors invite a ‘for-the-family’ orientation so that family will better understand how they may assist as their loved one to matriculate to graduation. The aforementioned deficiencies result in part to what is referred to as the ‘leaky pipeline’ [12], a term that describes the lack of progression of students into scientific careers requiring graduate degrees. Students often lose interest before completion of degree requirements and illustrates that during education from elementary school to a doctorate degree, underrepresented students drop off (‘leak’) where only a few ultimately end up in a professional scientific career that requires a graduate degree [12]. As explained, lack of information, not fully understanding the process, reduced salary projection, and family support all play a role in what is seen as the Leaky Pipeline [12]. However, to remain competitive, the US must recruit and train more underrepresented groups into math and science professions.

Gender diversity is just as salient as ethnic and racial diversity; it creates an environment of unique perspectives that can also augment the response to scientific inquiry [2,9,13,14,19,20,27–29]. While females are becoming more prominent in fields of science, unfortunately they still maintain an underwhelming minority. Paradoxically, the majority (61.7%) of bachelor degrees in 2018 were awarded to females [2]. However, two scientific fields in which females maintain that majority are the social sciences and psychology [2]. Approximately, 20% of females are represented in the fields of computer science and engineering; over 60% of females have bachelor degrees outside these fields. In terms of graduate degrees in science, the numbers are similar at 16.4%. The reasons for the deficiencies for females in science careers are a bit different. There are work interest and preferences that determine that females have a greater preference for social-oriented occupations that benefit society [30]. Secondly, there are work-family balance preferences where females are more likely to make sacrifices for family (i.e., there is overlap with in pursuit of graduate degree when females make plans to have children) that may prevent them from going forward [30]. Thirdly, gender based bias also contributes to the underrepresentation of females in science and math professions. Overt patterns of bias may be reduced, but such biases still exist and play a role in shaping female professional trajectories [30]. Thus, the Ernest Just Symposium was also developed to address these issues in medical education for underrepresented groups. The next part briefly reviews the extraordinary life of Dr. Ernest Everett Just, and the difficulties he overcame to pursue his dream of performing basic (hypothesis-driven) science research.

## Ernest Everett Just, Ph.D

Ernest Just was an African American born on 14 August 1883 in Charleston, SC. He believed in education, loved science, and lived a fascinating life. Ernest graduated from South Carolina State University at the age of 16 with a ‘*Licentiate of Instruction*’; in 1903, Mr. Just graduated from Kimball Union Academy with high honors; in 1907 he graduated from Dartmouth College (only African American male) Phi Beta Kappa, magna cum laude, with honors in botany, zoology, sociology and history [31]. It was at Dartmouth where he developed a strong curiosity for science and biology. Following Dartmouth, Mr. Just accepted a faculty position at Howard University and began performing research in 1909 after he was introduced to the Marine Biological Laboratory (MBL) by his mentor Dr. Frank Lillie, Ph.D. Mr. Just became so accomplished that he was awarded the first Spingarn Medal for Excellence in Academic Research given by the NAACP [31]. William Jeffrey, Ph.D., wrote in the *Biological Bulletin* ‘*Just was a genius in the design of his experiments.’* [32]. In fact, he obtained international acclaim for work at MBL and along with other scientists helped to transform the institution from a summer research retreat to a long-term high-profile research organization [31,33]. Ernest Just earned a Ph.D. in absentia from the University of Chicago (1916) with a thesis titled *Experimental Embryology* [31]. Dr. Lillie, who was Ernest Just thesis advisor comments on Dr. Just as a scientist ‘*His studies have been characterized not only by their care and precision, but also by a very considerable degree of scientific imagination. I do not think that any qualified zoologist would maintain that his work was not strictly first class*’ [31]. During his career, Dr. Ernest Just published more than 50 papers between 1912 and 1937, and wrote two books *Basic Methods for Experiments on Eggs of Marine Animals* and *Biology of the Cell Surface* [31]. The *Biology of the Cell Surface* is perhaps his most famous writing where he postulated a role for the cell surface in embryology, cellular development and evolution [34]. Overall, Dr. Just had a remarkable career of dedication, persistence, and love for science.

## The Ernest Everett Just Scientific Symposium

### Background and Goals

The Ernest Everett (EE) Just Symposium, celebrates the life and achievements of a pioneering African-American biologist, and presents an exciting opportunity for underrepresented students to further explore the variety of biomedical research and health professional career opportunities. The EE Just Symposium was first held in 1984 in Columbia, SC to celebrate the 100th anniversary of the birth of Ernest E. Just and honor his life’s work. The proceedings were published in 1985 with the title *The Cellular and Molecular Biology of Invertebrate Development*, and a dozen leaders in the field of invertebrate development were invited to present to scientists [35].

Since 2000, MUSC has hosted an annual EE Just symposium which has invited and exposed nearly 5,000 undergraduate, mostly underrepresented students, to careers in health science.

The goal(s) of the symposium, structured in a group environment, is to connect the past (EE Just) with present role models (faculty and students–peers) who can inspire students’ interest in science, educate on admission requirements, and to recruit into graduate science and medicine programs.

## Organization of symposium

### Methodology

The EE Just Symposium is an in-person one-day event that accommodates regional undergraduate institutions from South Carolina, North Carolina, Georgia, Maryland, and Florida. In addition, we also provide electronic live feeds of participation from institutions and individuals around the country. Undergraduate academic counselors at the institutions from the aforementioned states are contacted during the month of November before the symposium and the requests is to allow advanced (junior and senior) students to attend the EE Just Symposium. While seniors (and younger) in high school often attend, the majority are of college-age. A majority of the invited students are from underrepresented groups. We allow the advisor to determine which students attend.

The symposium is organized into six segments: (1) Impact of EE Just, (2) Role Models in Science and Medicine, (3) Panel Discussion, (4) Education Session, (5) Science Session, and (6) Table Conference.

The goal of the ‘*Impact of EE Just*’ segment is to develop an appreciation for Dr. Just’s research within the social context of the early 1900s and to understand the difficulties overcome by Dr. Just. Students learn about the life of Dr. Just, his contributions to scientific progress, and his determination to succeed. Embedded in this segment is the important concept of the history of science. As noted by Chamany, et. al., stories that focus on the people of biology remind students that biological research is a human endeavor and, like any other, is not isolated from politics, social norms, or the paradigms of the time [36]. For the symposium participants, this has proven to be a very inspiring component. The accomplished presenters for this segment are very familiar with the contributions that were made by Dr. Just at their respective institutions and/or organizations.

The goal of the ‘*Role Models in Science and Medicine*’ segment is to enlighten the students on scientific research by delineating the difficulties/benefits derived and the factors essential to succeed in science and medicine graduate curricula. Current role models in science and medicine are invited to give pearls of wisdom to the invited students. The role model presentations (Figure 3; demographics of presenters) are where speakers describe their scientific research and discuss the importance of keeping students engaged in the biomedical science career path, and examines an important health disparity (with an emphasis on investigator initiated research). Also within this segment is the presentation of the Ernest Everett Just Award for Excellence in Undergraduate Research. Prior to the symposium, submitted abstracts from the invited students are reviewed and the best are chosen for oral presentations. The winning students receive a certificate, an autographed copy of the *Black Apollo of Science; the Life of Ernest Everett Just* and a monetary award.

The next feature is the ‘*Panel Discussion*’ where the invited speakers’ field questions from the audience on a variety of subjects that interests the students. These speakers include the impact, role model, and science speakers from the program. Questions typically include how to prepare for graduate (science and medicine) school, how to decide on which graduate school, how to select a mentor, how to speak effectively to large audiences, and how to write manuscripts to targeted audiences. This segment also promotes direct communication between the invited students and the speakers. Interestingly, many of the questions are targeted towards the student winners of the EE Just award for Excellence in Undergraduate Research. Observations reveal that the winners of the undergraduate awards for excellence in research in the previous segment are identified as role models for the other students in attendance. They are bombarded with questions on how they became involved in research and how do they manage their respective daily schedules.

The ‘*Educational Sessions*’ are designed in part to inform invited students on the requirements for admission to the graduate program of their choice. Representatives from each college (Graduate Studies, Medicine, Nursing, Dental Medicine, Pharmacy, and Health Professions) will discuss the requirements for admission along with current students who can answer questions posed by the attending undergraduate students. Therefore, in an informal setting invited students have both faculty and student perspectives on the academic program of interest. In addition, invited students have the opportunity to tour the facilities in each of the aforementioned educational colleges.

The fifth segment is ‘*Science*’ and involves presentations from well-established national and international scientists on their respective research to the invited students and faculty. Such science presentations add a level of refinement to the symposium. Additions to this segment include a poster session highlighting the research of undergraduate students from participating colleges as well as current graduate students enrolled at MUSC and local colleges. This important component also promotes interactions among attendees. For invited undergraduate students, handouts are disseminated describing funding mechanisms for graduate education in areas of biomedical research from private foundations and public organizations like the National Institutes of Health (NIH). For example, the NIH funds grant programs such as Post Baccalaureate Research Education Program (PREP), Initiative in Maximizing Student Development (IMSD), diversity supplements to funded research grants, institutional training grants, and other relevant programs.

The final segment is the ‘*Table Conferences*.’ For this segment, students have conversations with facilitators and students from different institutions to discuss hopes and fears about the future of science and medicine along with health issues that affects them and their families. The surveys were given to the students at the end of the symposium; an average 80% of the attending students participated in the surveys. Self-reported data was measured using paired t-tests on a 10-point scale; p < 0.001 for both enthusiasm and interest in a research career. Males compared to female interest was measured on a 10-point scale with a p < 0.01, whereas undergraduate and K-12 were measured using p < 0.001. The EE Just Symposium was evaluated by an external evaluator commissioned by the South Carolina Experimental Program to Stimulate Competitive Research and Institutional Development awards. This included two PhD-level trained professionals in statistical data collection.

## Results

### Attendance and Outcomes of Previous Symposia (2013-2019) and demographics, participants and college major

The EE Just Symposium was evaluated for the years 2013–19, attendance for the EE Just Symposium has averaged 284 with a total of 1,992 total participants. In terms of the overall composition of the symposium, 73% were undergraduates and faculty advisors; 24% are K-12; 7% were faculty advisors to the students. Of the 1,992 participants for the aforementioned 7 years, 1488 were undergraduate students from regional four-year colleges and universities. Other participants included students sponsored by the Ernest Everett Just Foundation, Inc. (EEJFI), Empowering Minds Foundation, Inc., and the SC Area Health Education Consortium (AHEC). Both the EEJFI and Empowering Minds Foundation are based in the state of Maryland and dedicated to motivating middle, high school, and college age students into areas of STEM and members of the Omega Psi Phi fraternity, Inc. However, while members of the fraternity attend every year, neither of the aforementioned organizations attend every year. AHEC is dedicated to the education of students (high school and college) on science and on the importance of science education in the state of SC. In terms of gender, the majority of the attending students were female 74%. According to race, 73% of the participants were underrepresented groups; 23% were Caucasians; 1% Asians.

Of the 1,488 undergraduate students, 70% were biology majors, 13% were chemistry majors, and 6% were biochemistry majors. Several science majors were identified by students that include bioengineering, biotechnology, engineering, nursing and psychology. Moreover, many of the biology majors were also majoring in specific areas of science like pre-medicine, public health and chemistry. The participants were represented by 16 schools, 4 organizations; over 5 states, and the District of Columbia. Overall, the greatest number of participants traveled from institutions in South Carolina (56%), and the remaining numbers of participants traveled from the combined states of Maryland (12%), Georgia (11%), Florida (9%) and North Carolina (12%).

**Figure 1.**
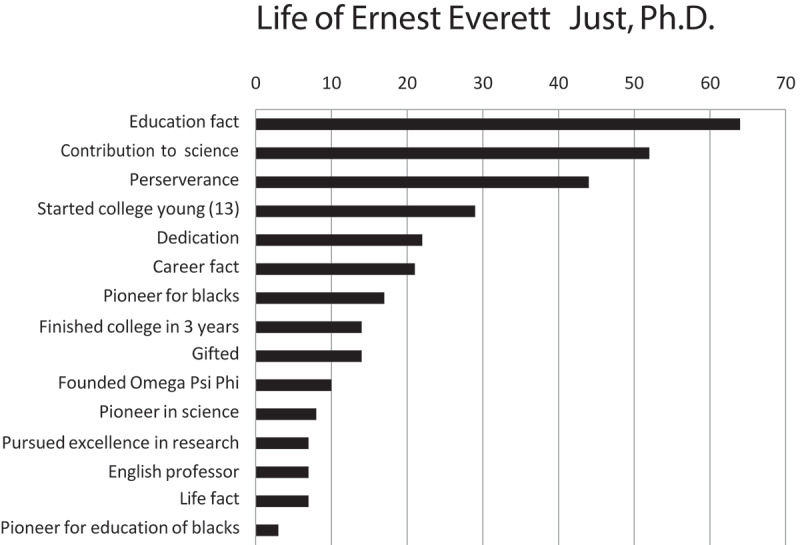
Education on the life of Ernest Everett Just, Ph.D. Participating students were questioned on the facts on aspects of the life and times of Ernest Just. The most common type of fact the participants describe was related to E.E. Just’s education; more than 25% of the participants described something such as ‘he went to my school’ or they named the universities Dr. Just attended; all of this was placed in the category of Educational Facts; Contribution to Science 52%; Perseverance 44%; Started college at 13 28%, Dedication 22%; Career facts 21% Pioneer for black 17%, finished college in 3 years 13%, Gifted 13%, Founder Omega Phi Psi 10%, Pioneer in science 8%, Pursued excellence in research 7%, English professor 7%, Life facts 7%, Pioneer for black 3%

**Figure 2.**
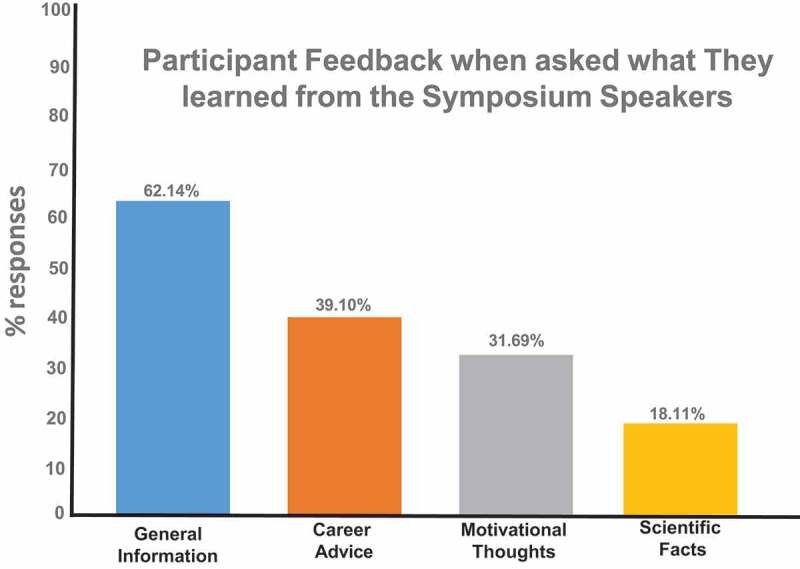
Participant feedback on what they learned from the symposium speakers. Participants were asked to record things that they learned from the speakers and the overriding answers were concerned with general information on graduate schools (62%); career advice; motivational thoughts; and scientific facts

Participants were asked to describe something they learned about EE Just and the results are shown in Figure 1. A majority of the students listed general information that they were not aware about Dr. Just. In terms of categories and other information from the other faculty presenters, students reported a better understanding of specific diseases like diabetes and hypertension that are more prevalent in African American populations. There was also career advice, motivational thoughts, and other scientific facts (Figure 2). Students were also asked to indicate on a scale of 1 to 10, the level of interest in pursuing an advanced degree both prior to and after the symposium according to their race. Participants were asked to indicate the level of enthusiasm on a scale of 1 to 10 in pursuing a career with research as an integral part of their professional work, again both prior to and after the symposium (Table 1). The overall increase in self-reported interest and enthusiasm was quite significant- interest in pursuing an advanced degree increased from a mean of 8.70 to 9.33 and enthusiasm for a career in research increased from 6.95 to 7.98. Students were previously interested in an advanced degree, but the symposium increased student interest (9.33/10.0). Further analysis was undertaken to determine potential differences based on gender, race, ethnicity and school level (K-12 and undergraduate).

**Figure 3.**
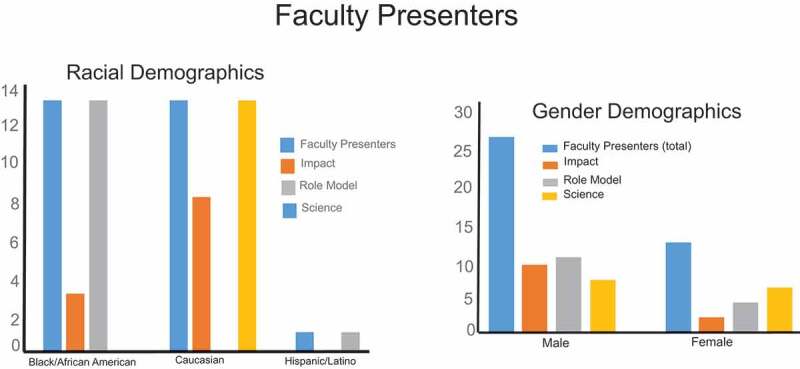
Demographics of the faculty role model, impact, and science speakers. The majority of the role model speakers were African American male. There were some female and Hispanic role model speakers. The science speakers Caucasian men and women

**Table 1.** The interest level was measured of responses of participants in pursing careers before and after the symposium by race on a scale of 1–10 (p < 0.005). In all three groups examined Caucasians, African Americans, and Hispanics the interest level in science as a career was increased. We measured the interest level of responses of participants in pursing careers before and after the symposium by gender and undergraduate, and K12. In all the examined categories the interest level in science as a career was increased after the symposium

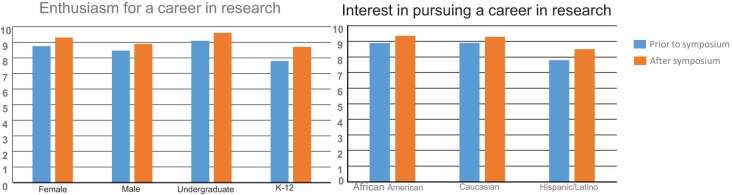

**Figure 4.**
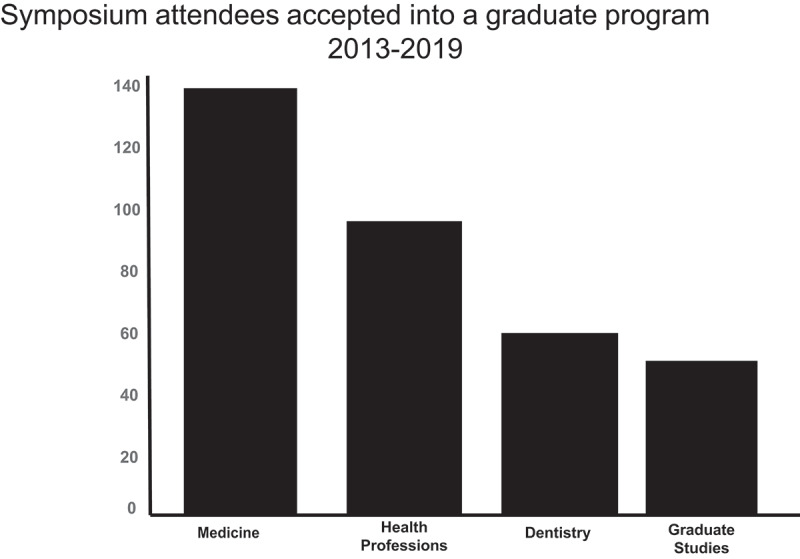
Symposium attendees accepted into graduate program at MUSC from 2013–2019 We monitor and track the student accepted into graduate programs, results show that we have Medicine 130; Health Professions 85; Dentistry 55 and Graduate studies 45

Females tended to have a higher interest in pursuing an advanced degree than the males, but this difference was not statistically significant. Males rated their enthusiasm for a career in research significantly higher than females, both prior to and at the end of the symposium (Table 1). There was no difference in how African Americans or Caucasian participants rated their interest in pursuing advanced degrees both before and after the symposium (Table 1). No differences based on ethnicity were found related to enthusiasm for a career in research. Similarly, K-12 students rated their interest in an advanced degree lower than undergraduate students, but there were no differences related to enthusiasm for a career in research for K-12 students (Table 1). In cooperation with advisors from the participating undergraduate schools, we were able to track students that enrolled at MUSC. Moreover, advisors from the participating undergraduate institutions provide tracking data that indicates in some capacity nearly 50% of the students who attended the EE Just Symposium have pursued graduate science and medicine education. In terms of recruitment into the medical university graduate programs, over 300 students who attended the 2013–2019 symposia have been accepted into graduate programs at the University (Figure 4).

## Discussion

Disparities in graduate medical education in the USA are a major problem. Thus, it is essential that medical educators invent creative ways to increase recruitment and ultimately the matriculation of all underrepresented groups. While undergraduate students interested in graduate medical education can locate information on admission requirements for institutions of higher learning, we believe that students receiving such information in a group setting has advantages over the individual research for several reasons. First, it sets the foundation for a singular focus (i.e., the energy of like-minded individuals) for the pursuit of graduate medical education [37, 38,#136]. Second, attending students can directly interact with personnel who are familiar with past medical and science heroes’ (like Dr. Just) contributions and directly converse with current role models in science and medicine outside of their institution [37,38,#136]. Third, from the table conferences, attending students tend the believethat this is their individual issue (i.e., no other students are experiencing these issues). However, attending the EE Just Symposium in a group setting allows the realization that students at other institutions are in the same situation with the similar fears and questions [37, 38,#136]. Fourth, visiting an institution in this manner allows the invited students to make lifelong contacts with faculty presenters and student peers [37,38]. Most importantly, using pass heroes to demonstrate to students that with knowledge of prerequisites and admission requirements, hard work, and perseverance they can earn a graduate degree in science/medicine. While Ernest Everett Just was born in Charleston, SC and has a connection to several previously mentioned institutions. Other past heroes that have connections to other institutions, maybe used in a similar manner, We also discovered three important outcomes from the symposium. First, Students can inspire each other as evidenced by the undergraduate presentations, which are one of the most popular segments. When they observe their peers performing hypothesis-based research, they become highly inspired. Second,attending students can mentor each other [38]. This was most evident at the table conferences where there was direct communication between students. This exposes the concept of social integration because the attending students have unique qualities that may and should be shared among attendees [39]. Third,from discussions with students and advisors there is a lack of knowledge on graduate science/medical education requirements that which is also observed in the literature [21,22]. This does not only include the academic prerequisites for admission, but also involves not understanding hypothesis driven research, choosing the appropriate academic program, and the important role of family. While it is not clear why the K-12 students rated their interest lower compared to the older students, one conclusion could be that the students are too young to benefit from the information (i.e., they are very far from graduate school). However, it is still clear that the symposium helped all groups to become more enthusiastic about a career in scientific research. More females tend to seek Ph.Ds. in science, especially in science and medicine, but females are still not as interested in a career in scientific research as their male counterpartsAlthough the Medical University cannot absorb 300–400 students/year into its’ graduate programs, we can make an impact by educating students on the qualifications/requirements necessary for acceptance into other university medical graduate programs (i.e., institutions have similar admission requirements). This coupled with the fact that the advisors from the undergraduate institutions report that 50% of the attendees will matriculate to agraduate program indicates that we are having asignificant impact. Either Dr.Just or other famous scientist that either attended the institution or have some institutional connection may be used. The paradigm of linking past and present heroes can serve as amodel for other universities, research institutes and organizations.

## Conclusion

While inequalities in the numbers of minorities working in science and math careers will not improve overnight, activities like the *Ernest Everett Just Scientific Symposium* provide a working model that can be duplicated at other institutions. The challenges encountered by scientists in the past (like Dr. Just) and present day students and scientists are not vastly different from today. This includes the balancing of school, work, home, writing papers, choosing the right mentors, conscious and unconscious bias, and the constant search for research funding. Dr. Just’s life serves as an outstanding example of curiosity coupled with persistence to overcome a number of obstacles on the path to become a very successful scientist. Using the life and times of Ernest Everett Just, Ph.D. or other famous scientists to inspire, educate, and recruit students into the graduate biomedical workforce is a pathway that may begin to eliminate racial disparities in our universities and health-care institutions.
